# Functional Nutrients for Epilepsy

**DOI:** 10.3390/nu11061309

**Published:** 2019-06-10

**Authors:** Ji-Eun Kim, Kyung-Ok Cho

**Affiliations:** Department of Pharmacology, Department of Biomedicine & Health Sciences, Catholic Neuroscience Institute, Institute of Aging and Metabolic Diseases, College of Medicine, The Catholic University of Korea, Seoul 06591, Korea; jonin12@naver.com

**Keywords:** epilepsy, seizure, nutrients, omega-3 fatty acid, vitamin D3, vitamin E, vitamin B6

## Abstract

Epilepsy is a common neurological disorder of which seizures are a core symptom. Approximately one third of epileptic patients are resistant to antiepileptic drugs and therefore require alternative therapeutic options. Dietary and nutritional supplements can in some cases replace drugs, but with the exception of ketogenic diets, there are no officially recommended dietary considerations for patients with epilepsy. In this review we summarize a selection of nutritional suggestions that have proved beneficial in treating different types of epilepsy. We describe the types of seizures and epilepsy and follow this with an introduction to basic molecular mechanisms. We then examine several functional nutrients for which there is clinical evidence of therapeutic efficacy in reducing seizures or epilepsy-associated sudden death. We also discuss experimental results that demonstrate possible molecular mechanisms elicited by the administration of various nutrients. The availability of multiple dietary and nutritional candidates that show favorable outcomes in animals implies that assessing the clinical potential of these substances will improve translational medicine, ultimately benefitting epilepsy patients.

## 1. Introduction

Epilepsy is a common neurological disease with a prevalence of approximately 0.4 to 1% which affects people of all ages and races, and both genders [1]. It is a heterogeneous group of disorders with a variety of etiologic backgrounds associated with seizure manifestation [2]. Germline or somatic mutations; structural brain lesions including stroke, cancer, trauma, and status epilepticus; various infections; metabolic defects; and autoimmune-mediated dysregulation of inflammation can all elicit seizures, which range from altered sensation of the five senses to rapid head and eye movement to brief or prolonged loss of consciousness to uncontrolled muscle contractions and spasms [1,3]. Vigorous efforts to delineate different types of epilepsy in a more precise manner and to treat patients using antiepileptic drugs with appropriate mechanisms of action have made great progress in suppressing unexpected seizures. Some 70% of epilepsy patients now exercise seizure management [1]. However, for the remaining 30%, who suffer from intractable epilepsy, treatment options are limited to different multi-drug therapies or surgery if applicable. Alternative treatments for patients with refractory epilepsy are therefore needed. 

Functional foods and bioactive nutrients can be considered part of adjunctive therapy or an essential strategy for the treatment of epilepsy, depending on the etiologic nature [4,5,6,7,8,9,10,11,12,13,14,15,16,17,18,19]. For certain metabolic epilepsies, supplying deficient nutrients can correct inborn errors and stop seizures [20], while for most refractory epilepsies, no obvious effective dietary recommendations are available other than ketogenic diets [21,22]. To provide comprehensive information about nutritional supplements for the treatment of epilepsy, we summarize here functional nutrients that have shown therapeutic efficacy in drug-refractory epilepsy. First, we provide an overview of epilepsy and its essential molecular mechanisms. We then summarize a selection of biologically active nutrients that can be used as add-on or first-line therapy for intractable epilepsies, based on PubMed search criteria with combinations of keywords including “nutrient”, “functional food”, “nutrition”, “nutritional”, “dietary supplement”, “supplementation”, “vitamin”, “mineral”, “fatty acids”, “epilepsy”, “epilepsy syndrome”, “refractory epilepsy”, “intractable epilepsy”, “drug-resistant epilepsy”, “temporal lobe epilepsy”, “genetic generalized epilepsy”, and “metabolic epilepsy”, etc. Finally, we discuss the experimental evidence supporting the clinical findings.

## 2. General Overview of Epilepsy 

### 2.1. Classification of Seizures

**Figure 1:**
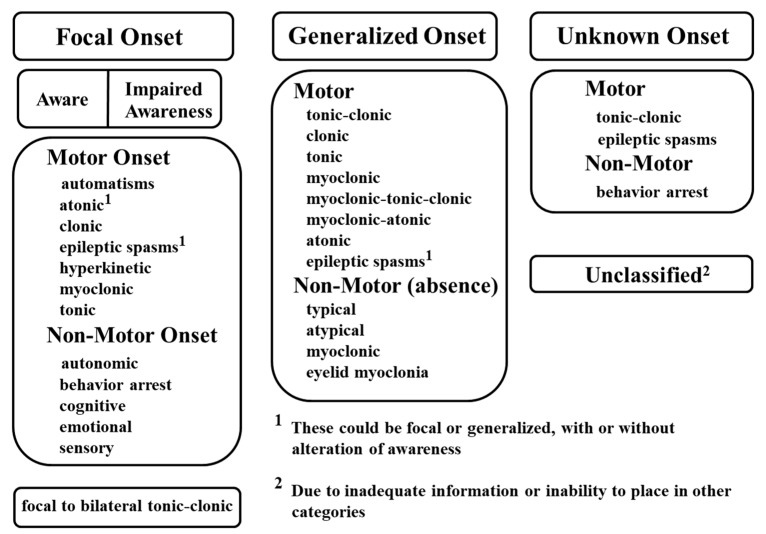
International League Against Epilepsy (ILAE) guidelines for the classification of seizure types. Reprinted with permission from Wiley [23].

A variety of factors can elicit the transient, synchronous, and excessive neuronal activities that cause seizures. Because current knowledge is not yet sufficient to distinguish between various types of seizures based on etiology, seizures are classified by their mode of onset, degree of awareness, and initial symptoms. In the latest classification released by the International League Against Epilepsy (ILAE) in 2017 (Figure 1), seizure types are divided into three groups: focal onset, generalized onset, and unknown onset [23]. Focal and generalized seizures are classified by where and how seizure activity begins. For example, focal onset seizures are localized in discrete areas of the brain, usually in one hemisphere, whereas generalized onset seizures immediately engage bilaterally-distributed networks of the brain. For focal seizures, the level of awareness can be further described to display seizure characteristics, in addition to the first prominent sign such as motor symptoms (automatisms, atonic, clonic, epileptic spasms, hyperkinetic, myoclonic, tonic) or non-motor symptoms (autonomic, behavior arrest, cognitive, emotional, sensory). Finally, focal to bilateral tonic-clonic seizures are categorized as focal seizures when an initial seizure focus is identifiable. Generalized onset seizures can be divided into motor or non-motor (absence) seizures. Depending on the clinical diagnostic observations, motor seizures can be further classified as tonic-clonic, clonic, tonic, myoclonic, myoclonic-tonic-clonic, myoclonic-atonic, atonic, and epileptic spasms, while non-motor seizures can be categorized as typical, atypical, myoclonic, and eyelid myoclonia. Seizures of unknown onset can be described as “unclassified” or with additional characteristics such as motor (tonic-clonic or epileptic spasms) or non-motor (behavior arrest) symptoms. However, if seizure onset can be determined at a later time, seizures of unknown onset can be re-categorized as either focal or generalized seizures. 

### 2.2. Classification of Epilepsy

**Figure 2:**
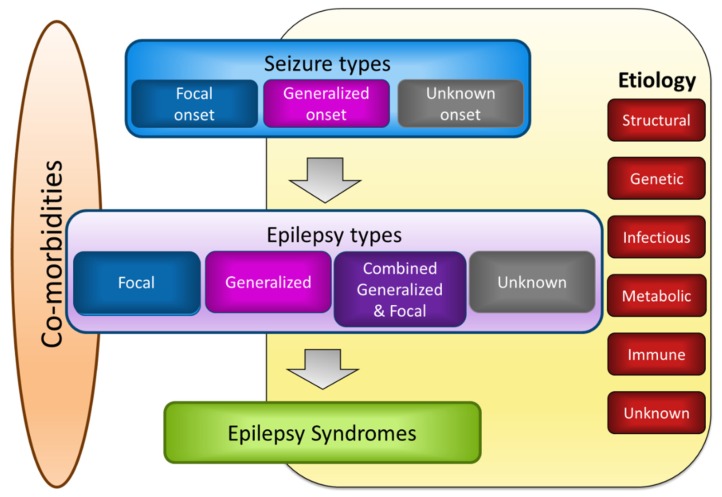
ILAE guidelines for the classification of epilepsies. Reprinted with permission from Wiley [3].

Epilepsy has been defined as the experience of at least two unprovoked seizures more than 24 h apart. However, ILAE has revised the definition of epilepsy, providing wider diagnostic criteria that include additional conditions: (1) one unprovoked seizure and a 60% or greater probability of further seizures occurring over the next 10 years, which is similar to the general recurrence risk after two unprovoked seizures; (2) a diagnosis of epilepsy syndrome [2]. As epilepsy includes heterogeneous encephalopathies sharing a common symptom, namely, seizures, continuous efforts to categorize epilepsies based on their characteristics have been made [3,24]. The revised classification proposes three levels of diagnostic steps with an additional two factors (Figure 2) [3]. The first level involves determining seizure types that have been described in the previous section. Once the seizure types can be classified with clinical tools, such as an electroencephalogram (EEG) and proper imaging modalities, the epilepsy types can be further determined. The four groupings for the second level classification of epilepsies are focal, generalized, combined generalized and focal, and unknown. Focal epilepsies generally show abnormal EEG activity in one hemisphere, whereas generalized epilepsies show generalized spike-wave activity in both hemispheres. However, epilepsy can include multiple types of seizures. For instance, generalized epilepsy can manifest as tonic-clonic seizures and myoclonic jerks. The third diagnostic level involves evaluating whether a patient’s epilepsy can be recognized as a distinctive clinical disorder, i.e., epilepsy syndrome. Although there is no official ILAE list of epilepsy syndromes, Ohtahara syndrome, early myoclonic encephalopathy, West syndrome, Dravet syndrome, Lennox-Gastraut syndrome, Lindau-Kleffner syndrome, childhood absence epilepsy, juvenile absence epilepsy, juvenile myoclonic epilepsy, generalized tonic-clonic seizures alone, Rasmussen syndrome, Sturge-Weber syndrome, mesial temporal lobe epilepsy (TLE) with hippocampal sclerosis, self-limited epilepsy with centrotemporal spikes, self-limited occipital epilepsies of childhood, and Panayiotopoulos syndrome can be included within epilepsy syndrome, and are differentially classified based on the age of onset, seizure triggers, and diurnal variation, together with EEG and imaging findings.

The type of epilepsy can be further specified by etiological implication, which can be of structural, genetic, infectious, metabolic, immune, or unknown etiology. Structural abnormalities such as stroke-induced infarction, trauma, hippocampal sclerosis, and hypothalamic hamartoma are associated with an increased risk of being epileptic. Brain lesions visualized by imaging tools can also identify candidate surgical areas for the treatment of acquired epilepsy. As basic epilepsy research and genetic screening techniques have improved more than 400 genetic mutations associated with epilepsy have been identified, including SCN1A, SCN2A, SCN3A, SCN8A, SCN9A, SCN1B, KCNQ2, KCNQ3, KCTD7, CACNA1H, CLCN2, GABRA1, GABRB3, GABRG2, SLC2A1, CDKL5, ARX, CHD2, STXBP1, and PNPO [25,26,27]. “Genetic” does not mean “inherited,” as both germline and de novo mutations can cause epilepsy. Diverse infectious organisms can cause epilepsy, and these forms are different from seizures originating from simple acute meningitis or encephalitis. Examples include neurocysticercosis, tuberculosis, human immunodeficiency virus (HIV), cerebral malaria, cerebral toxoplasmosis, and congenital Zika or cytomegalovirus infections. Metabolic epilepsy refers to metabolic diseases that manifest unprovoked seizures. Most metabolic epilepsies, such as porphyria, uremia, aminoacidopathies, and pyridoxine deficiency, have a genetic basis, but acquired metabolic epilepsy, i.e., cerebral folate deficiency, also exists. Epilepsies with immunological backgrounds have increased due to the advancement of immunological tests that utilize numerous antibodies. Such epilepsies are essentially variants of autoimmune encephalitis, including anti-N-methyl-D-aspartate (NMDA)-receptor encephalitis or anti-leucine-rich glioma-inactivated-1 (LGI1) encephalitis, in which autoantibodies for autoantigens elicit excessive inflammation in the brain.

The final diagnostic step is the consideration of comorbidities associated with epilepsy. Frequently listed abnormalities include learning and memory impairment, mood disturbance, autism spectrum disorders, and psychosocial issues. However, as epilepsies cover a range of seizure-related disorders, more severe comorbidities that can cause permanent sequelae such as cerebral palsy, gait disturbance, or developmental delay can also be observed. Accurate categorization will therefore be essential for the comprehensive treatment and better understanding of the complex nature of epilepsy.

## 3. Molecular Mechanisms of Epilepsy

An imbalance between excitation and inhibition has long been thought to be one of the main mechanisms that cause spontaneous seizures, based on the classical channelopathy model of epilepsy [27,28]. Single-nucleotide mutations such as missense, frameshift and nonsense mutations, copy number variations by de novo or inherited DNA deletion or duplication, and chromosomal copy number abnormalities can cause genetic epilepsies [29]. However, acquired epilepsy (i.e., TLE) can develop from the initial insult and subsequent accumulation of multiple structural changes that promote network excitabilities, ultimately ending in spontaneous recurrent seizures [28]. In addition, rapid technological advancements in gene panel screening and next-generation sequencing for both genetic and acquired epilepsies have identified several new molecular candidates that do not appear to have a direct link with network hyperexcitability [25,29,30]. Emerging evidence suggests that the pathophysiological mechanisms of epilepsy are much more complicated than expected, prompting demand for more vigorous research. In this review, we discuss three basic mechanisms that are commonly observed in both genetic and acquired epilepsies. 

### 3.1. Ion Channel and Neurotransmitter Dynamics in Epilepsy

Many voltage- and ligand-gated ion channels are dysregulated in epilepsies caused by both genetic mutations and structural abnormalities [26,27,31]. Active exome sequencing can uncover a variety of mutations in sodium, potassium, and calcium channel genes, in addition to gamma-aminobutyric acid (GABA) and NMDA receptor genes [26,27]. We will cover the critical facts concerning some exemplary channel mutations in this review; for more detailed information, refer to [26,27]. Voltage-dependent sodium channels such as SCN1A, SCN2A, SCN3A, SCN8A, SCN9A, and SCN1B, which encode Na_v_1.1, Na_v_1.2, Na_v_1.3, Na_v_1.6, Na_v_1.7, and Na_v_β1, respectively, have had many different mutation sites reported for each gene, including both nonsense and missense mutations [26]. This may explain why patients with the same mutation can exhibit variable seizure severity. Moreover, as multiple DNA regions can be mutated within the same gene, it is likely that the SCN1A mutation can be identified in multiple kinds of genetic epilepsies, including severe myoclonic epilepsy of infancy, epilepsy with febrile seizures plus, and partial epilepsy with febrile seizures plus. Although the details of the sodium channel dysfunction differ depending on the individual mutation site, overall changes are made toward channel hyperactivity, promoting neuronal hyperexcitation [32,33,34,35]. This explanation is supported by knockout animals showing seizure phenotypes [36,37,38,39,40]. Finally, SCN1A and SCN8A mutations have been associated with sudden unexpected death in epilepsy (SUDEP). Although the etiologic mechanisms behind SUDEP are unclear, the association between refractory seizures by SCN1A and SCN8A mutations and disrupted cardiorespiratory function has been proposed as a risk factor for SUDEP [34,41,42,43,44,45]. 

With regard to potassium channels, KCNQ2 and KCNQ3 mutations are frequently observed in patients with self-limited familial neonatal seizures [46]. KCNQ2 and KCNQ3 encode voltage-gated potassium channels that produce the M current, inducing a hyperpolarizing shift in membrane voltage. Loss of their function can increase neuronal hyperexcitability, leading to spontaneous seizure activities as reported in KCNQ2 and KCNQ3 knockout mice [47]. Among calcium channel mutations, CACNA1H, a T-type calcium channel subunit called Ca_v_3.2, has shown three missense mutations in patients with genetic generalized epilepsy and has displayed enhanced channel function [48,49]. As for ligand-gated ion channels such as GABA_A_ and glutamate NMDA receptors, GABRA1, GABRB3, and GABRG2 mutations encoding α1, β3, and γ2 subunits of GABA_A_ receptor, and GRIN1, GRIN2A, GRIN2B, and GRIN2D mutations encoding NR1, NR2A, NR2B, and NR2D subunits of NMDA receptors, respectively, have been identified in genetic epilepsy [27]. Similarly to the other channel mutations described above, most mutations of GABA_A_ receptors cause loss of function, whereas mutations of NMDA receptors induce the gain of function of NMDA receptors [27], anticipating network hyperexcitability in these genetic epilepsies. 

Ion channel abnormalities have also been examined extensively in acquired epilepsy, including TLE [31]. In general, individual perturbations of many different ion channels occur toward the facilitation of network hyperexcitability. For example, transcription of the T-type calcium channel Ca_v_3.2 was shown to be upregulated after pilocarpine-induced status epilepticus [50], which can change the mode of neuronal firing from regular to intrinsic burst firing after acute seizures [51,52], strengthening the propensity of neuronal excitation. Other important players mediating abnormal neuronal transmission in TLE are ligand-gated GABA and glutamate receptors. Failure of GABAergic inhibition contributes to the development of epilepsy [53,54]. It may accompany enhanced excitatory transmission by molecular re-assembly of α-amino-3-hydroxy-5-methyl-4-isoxazolepropionic acid receptor subunits, as GluR2 transcription has been shown to decrease after status epilepticus [55], suggesting a possible increase in calcium permeability through this receptor [56]. Alterations of GABA_A_ receptor subtypes in TLE can also participate in compensation of the seizure-induced imbalance between excitation and inhibition, contributing to complex epileptogenesis [57].

### 3.2. Oxidative Stress in Epilepsy

Reactive oxygen species (ROS) contain unpaired electrons. Common free radicals generated in cells include superoxides, hydroxyl radicals, and hydrogen peroxide. These ROS can be produced as a byproduct of physiological mitochondrial respiration [58] or the activation of cellular enzymes such as nitric oxide synthase, cyclooxygenase (COX), lipoxygenase, xanthine oxidase, cytochrome P450, and nicotinamide adenine dinucleotide phosphate oxidase [59]. Numerous experimental studies have reported excessive ROS production in epilepsies that exceeds the capability of endogenous antioxidants such as superoxide dismutase (SOD) and thioredoxin reductases [60]. The resulting oxidative stress after seizure activities can lead to peroxidation of cellular macromolecules such as proteins, lipids, and DNA. These alterations undermine cellular function in general because protein oxidation can change the structure and activity of crucial enzymes such as sodium-potassium ATPase [61]. ROS-induced alteration of sodium-potassium ATPase can then impair the efficient reversal of neuronal depolarization-induced electrochemical gradients, causing excitotoxicity. In addition, peroxidation can alter the properties of lipid bilayers, influencing membrane permeability and the activity of membrane proteins, including ion channels, and ligand-gated neurotransmitter receptors [62]. Together, excessive ROS-mediated oxidative stress induced by seizures can predispose neuronal hyperexcitability.

Some genetic epilepsies exhibit mitochondrial DNA mutations affecting complex I of an electron transport chain [63,64]. Intractable myoclonic epilepsy with ragged-red fibers (MERRF) and Leigh syndrome have displayed increased ROS production due to complex I dysfunction [65,66]. When the MERRF gene is mutated, impairments of mitochondrial calcium uptake and ATP synthesis, which can trigger cellular energy deficiency-initiated neuronal excitotoxicity, are observed [67]. Moreover, a thioredoxin reductase 1 mutation reported in patients with genetic generalized epilepsy can increase sensitivity to hydrogen peroxide challenges [68], implying insufficient ROS detoxification. Another study has identified patients with Lafora disease as showing progressive myoclonic epilepsy and the mutation of glucan phosphatases EPM2A and EPM2B [69,70]. Fibroblasts obtained from Lafora disease patients have been shown to exhibit increased production of ROS, including mitochondrial superoxide, along with a reduction in mitochondrial SOD and catalase expression [70]. This has been further supported by both EPM2A and EPM2B knockout mice exhibiting reduced SOD activity in addition to increased levels of lipid peroxidation, suggesting defective cellular processes that reduce oxidative stress by genetic mutations. These pieces of evidence collectively support the idea of elevated oxidative stress in the pathophysiology of various genetic epilepsies.

In acquired epilepsy syndromes, multiple reports have consistently shown that seizures induce oxidative damage following excessive ROS generation in the brain, exacerbating network excitability [59,60,71]. Various markers of protein and lipid peroxidation have been reportedly increased in surgically-resected human TLE tissues [72,73,74,75], despite upregulated glutathione and SOD antioxidant systems [73,76,77], suggesting seizure-induced oxidative damage. Animal studies have also confirmed increased ROS production in the hippocampus in association with oxidative damage to proteins, lipids, and mitochondrial DNA [78,79,80,81,82,83]. A cause-and-effect relationship between oxidative stress and the development of epilepsy has been suggested by reports that spontaneous recurrent seizures in SOD mutant mice are correlated with augmented oxidative damage and neuronal death due to insufficient antioxidant expression [84,85]. In summary, these results emphasize the important role of oxidative stress in epileptogenesis and related neuropathology.

### 3.3. Inflammation in Epilepsy

The majority of genetic-epilepsy-associated genes studied to date involve ion channels and a few strands of mitochondrial DNA. Little has been reported with respect to inflammatory cytokines and enzymes. However, two papers investigating single-nucleotide polymorphisms of interleukin 1β (IL-1β) in patients with febrile seizures have raised the possibility of de novo mutations [86,87]. They found that IL-1β TT genotypes were over-represented in febrile seizure–experiencing groups compared with healthy controls. Moreover, serum IL-1β concentration was significantly higher in patients experiencing febrile seizures, suggesting that an IL-1β polymorphism may promote proinflammatory conditions after febrile seizures and contribute to the development of TLE in a chronic phase.

In the hippocampus, activation of astrocytes and microglia has been consistently observed after acute seizures in multiple types of epilepsy, including TLE, tuberous sclerosis complex, traumatic brain injury, Rasmussen syndrome, and focal cortical dysplasia [88,89]. Activated glial cells can release various inflammatory mediators, including IL-1β, high mobility group box-1 (HMGB1), interleukin 6 (IL-6), tumor necrosis factor α (TNF-α), COX-2, and complementary factors after acute seizures [90]. Glial cells respond rapidly to seizure activities as cytokine levels in the brain increase within 30 min of seizure onset and stay at high levels even in chronic epileptic stages [88]. With regard to the many inflammatory mediators released in epileptogenic brains, the pro-epileptic roles of IL-1β are well established. Upregulated IL-1β and its inhibition by interleukin 1 receptor agonist have been found to facilitate and alleviate the induction of status epilepticus, respectively, with the regulation of glutamatergic neurotransmission [91,92]. Moreover, proinflammatory cytokines increase blood–brain barrier (BBB) permeability, supporting dynamic relationships among BBB breakdown, inflammation, and reactive glial activation [91]. HMGB1, another pro-epileptic mediator that can be secreted by glial cells and dead neurons, acts through a toll-like receptor (TLR) system [93]. Increased expression of HMGB1 and TLR4 is seen in TLE and focal cortical dysplasia [93]; HMGB1 can enhance both acute and chronic seizure susceptibilities in a TLR4-dependent manner [94]. 

IL-6 can be anti-epileptic, and, as experimental and clinical evidence has shown, it is involved in glutamatergic receptor-mediated acute seizure aggravation [95,96]. However, TNF-α can serve either pro- or anti-epileptic roles, depending on the context. For example, TNF-α infusion into the lateral ventricle can increase seizure susceptibility [97], supporting the pro-epileptogenic effects of TNF-α. Moreover, TNF-α has demonstrated a pro-convulsive effect involving activation of TNF-α receptor 1 but an opposite anti-ictogenic effect has also been observed when TNF-α signaling is mediated by TNF-α receptor 2 [98]. In support of the anticonvulsive effects of TNF-α signaling pathways, knockout of TNF-α and its receptor p75 using a genetic approach has shown exacerbated seizures with aggravated cell death [99]. 

Multiple proinflammatory and anti-inflammatory mediators secreted by activated glial cells can play multifaceted roles in various types of epilepsy, affecting the network excitability. As their modes of action are complex and intricate, a deeper appreciation of the underlying mechanism will provide valuable insights into the complicated pathophysiology of epilepsy. 

## 4. Functional Nutrients Beneficial in Epilepsy

A variety of nutritional interventions have been carried out in patients with epilepsy to investigate the clinical significance of functional nutrients in the control of seizures. Despite ample experimental evidence demonstrating the therapeutic potential of various nutrients, clinical data assessing the anticonvulsive effects of nutritional supplements are scarce. In this section we introduce several nutrients that have demonstrated beneficial effects in multiple clinical studies and explore their possible molecular mechanisms.

### 4.1. Omega-3 Polyunsaturated Fatty Acids

Omega-3 polyunsaturated fatty acids (n-3 PUFAs) are essential fatty acids that cannot be synthesized in sufficient quantities in the body. They must therefore be obtained from the diet or supplements. Marine fish (salmon, tuna, and mackerel), nuts and seeds (flaxseed, chia seeds, almonds, and walnuts) are rich in dietary n-3 PUFAs [100]. The three major omega-3 fatty acids are docosahexaenoic acid (DHA) and eicosapentaenoic acid (EPA), which are found mainly in marine fish oils, and alpha-linolenic acid (ALA), which is the main component of plant oils. Among these three, DHA is the primary n-3 PUFA in the brain and constitutes 10–20% of total brain fatty acids, whereas ALA and EPA constitute less than 1% [101]. DHA can serve as a structural component of neuronal membranes, modulating membrane biophysical properties [102], ion channel functions [103], and neurotransmitter signaling [104].

The therapeutic benefits of omega-3 fatty acids for epilepsy have been anticipated, as n-3 PUFA can reduce cardiac arrhythmia, which involves the hyperexcitability of cardiac cells [105]. The first clinical, open-label study of the therapeutic potential of fish oil in patients with epilepsy found an anticonvulsive effect associated with n-3 PUFA [4]. However, subsequent clinical studies have produced more controversial results (Table 1). For example, while some randomized clinical trials have found that consuming approximately 0.6–2 g of fish oil reduces seizure frequency and duration [5,6,7,8,9], other small-scale, non-randomized studies have reported no regulatory efficacy for seizures [10,106,107,108,109]. Despite several animal studies suggesting omega-3 fatty acids suppress seizures, whether n-3 PUFA has significant benefits regarding the control of seizures remains unclear. Still, considering the goal of identifying nutritional supplements that assist drug therapy, and given the established safety of fish oil at dosages of less than 4 g per day, fish oil can be recommended for the regulation of seizure generation [110]. 

A consequential issue in addition to seizure control is SUDEP, a leading cause of death in patients with intractable epilepsy [111,112]. Based on the strong beneficial roles of n-3 PUFAs in the attenuation of arrhythmia and sudden cardiac death [105,113], it is plausible that fish oil may promote cardiac health. Many researchers have tried to link n-3 PUFA to the prevention of SUDEP via the reduction of cardiac arrhythmias [114,115,116,117]. A randomized double-blind study assessing 11 patients with intractable epilepsy provided initial evidence that fish oil supplementation could restore heart rate variability (HRV) in a subgroup with very low baseline HRV, which can be a marker of sudden death risk [10]. However, as this is the only study implicating n-3 PUFA supplementation in SUDEP prevention, more efforts are warranted before drawing conclusions regarding the efficacy of omega-3 fatty acids in epilepsy-associated mortality.

An elevated seizure threshold associated with n-3 PUFAs has been verified in a variety of animal models [103,118,119,120,121,122,123,124]. With regard to the possible underlying molecular mechanisms of n-3 PUFA-mediated anticonvulsive effects, the regulation of ion channel activities, as well as anti-oxidative and anti-inflammatory effects, has been proposed. For example, PUFAs can inactivate neuronal voltage-dependent sodium and calcium channels [125,126]. Moreover, DHA, ALA, and EPA treatment of mouse dorsal root ganglion neurons reportedly facilitates the opening of potassium M-channels, dampening neuronal activity [127]. DHA can also modulate ionotropic GABA_A_ receptors, alleviating neuronal hyperactivities [104,128,129,130]. Increasing inhibitory potentials by n-3 PUFA-associated ion channel modulation can result in improved survival of GABAergic interneurons and excitatory pyramidal neurons in the hippocampus after pilocarpine-induced status epilepticus, as n-3 PUFAs may stabilize seizure-induced hyperexcitable states [131,132]. Another possible mechanism is the anti-oxidative effect of omega-3 fatty acids. Using the pentylenetetrazole model of epilepsy, several researchers have demonstrated that n-3 PUFA administration increases SOD, catalase, and glutathione peroxidase activity and expression [122,124,133]. This relationship has been further supported by a different model of epilepsy, reinforcing the anti-oxidative function of n-3 PUFAs [134]. When a recent randomized clinical trial assessed cytokine levels after 16 weeks of n-3 PUFA treatment, TNF-α and IL-6 concentrations were found to significantly decrease in the supplemented group compared to the placebo group [9]. In the pilocarpine model of epilepsy, proinflammatory cytokines in the blood of omega-3 fatty acid-treated rats, including IL-1ß, TNF-α, and IL-6,all decreased [135], supporting the anti-inflammatory role of n-3 PUFAs in epilepsy.

### 4.2. Vitamin D3 (Cholecalciferol)

Vitamin D is a fat-soluble vitamin that comes in multiple forms, including vitamin D2 (ergocalciferol) and D3 (cholecalciferol). Among these, vitamin D3 is naturally present in animals and can be synthesized by exposure to sunlight. However, vitamin D can also be obtained from dietary sources such as oily fish (cod, swordfish, salmon, tuna, and sardines), dairy products (milk, cheese, and yogurt), meat (beef liver), and mushrooms [136]. Once vitamin D is produced or ingested, the liver converts it to 25-hydroxyvitamin D (25-OH vitamin D, calcidiol). The kidney then makes the active form, 1,25-dihydroxyvitamin D (1,25-[OH]_2_ vitamin D, calcitriol) [137]. Vitamin D3 maintains bone integrity by promoting the absorption of calcium and phosphorus. In the brain, vitamin D is a neuroactive steroid, acting through nuclear or membrane vitamin D receptors [138]. Functions of vitamin D in the central nervous system include regulation of neurotransmitters, neuronal differentiation, axonal growth, voltage-sensitive calcium channels, neurotrophic factors, and ROS [138], which can affect proper neuronal function. 

Several studies have reported that both young and adult patients with epilepsy show vitamin D deficiency in their blood [139,140,141], providing a rationale for giving vitamin D to people with epilepsy. To date, three clinical studies have investigated the effects of vitamin D on seizure control (Table 2) [11,12,13]. The first study recruited 23 epileptic patients who were divided into two experimental groups, with one given 4000 international units (IU) of vitamin D3 daily for 28 days and 16,000 IU of vitamin D3 for the next 28 days, followed by an initial observation period, and the other given the placebo first and then 8000 IU of vitamin D3 for each of the 28-day periods [11]. In patients who received vitamin D3, the mean seizure frequency was significantly reduced to 67–71% of that of the baseline, suggesting an anticonvulsive effect. A similar observation has reported in another clinical study in which correction of vitamin D3 deficiency reduced seizure frequency by up to 40% for drug-resistant epilepsy [12]. A recent cross-sectional cohort study which assessed 160 patients with epilepsy, however, has reported conflicting results which have shown no therapeutic efficacy of vitamin D3 on control of seizure generation [13]. More elaborately designed studies with larger sample sizes will be required to resolve question of the anticonvulsive effects of vitamin D3 with regard to epilepsy. 

Because vitamin D3 has versatile therapeutic efficacies on cardiovascular diseases [142], vitamin D3 may have an impact on SUDEP [143]. Although there is no direct evidence demonstrating a possible role for vitamin D in SUDEP prevention, one group has reported that vitamin D supplementation can modulate HRV [144,145,146,147], which is frequently lower in patients with drug-refractory epilepsy, a group at high risk of SUDEP [148,149]. This raises the possibility that vitamin D may have therapeutic benefits for the prevention of SUDEP. 

Animal models of epilepsy have also indicated anticonvulsive effects from vitamin D treatment [150,151,152,153]. When the vitamin D receptor has been genetically deleted, seizure susceptibility has been significantly augmented in epileptic mice [154]. Possible mechanisms of vitamin-D3-mediated anticonvulsive effects include a reduction in voltage-sensitive calcium channel expression [155,156] via the attenuation of neuronal calcium overload after seizure activities. Vitamin D can also inhibit the generation of inducible nitric oxide synthase, which relieves oxidative cellular damage by blocking nitric oxide production [157]. In addition, the immunomodulatory action of vitamin D has been suggested, as vitamin D can suppress various cytokines, including interferon (IFN)-γ, IL-2, TNF-α, and IL-6 [158,159]. We have also reported that an edible mushroom, *Hericium erinaceus*, in which ergosteol can be converted to vitamin D [160], may protect hippocampal neurons against pilocarpine-induced status epilepticus by decreasing COX-2-expressing glial cells [161]. The idea of the anti-inflammatory properties of vitamin D has been further supported by a clinical study that reported how concentrations of IL-1β, IL-6, interleukin-8, macrophage inflammatory protein 1β, monocyte chemoattractant protein-1, and IFN-inducible protein 10 markedly decreased after co-treatment with vitamins D and B12. In summary, vitamin D appears to be a promising candidate that will complement epilepsy treatment with antiepileptic drugs. 

### 4.3. Vitamin E

Vitamin E refers to a group of fat-soluble antioxidants [162]. Vitamin E consists of eight vitamer chemicals with similar molecular structures and functions, namely, four tocopherols (α-, β-, γ-, and δ-tocopherols) and four tocotrienols (α-, β-, γ-, and δ-tocotrienols). Among these, α-tocopherol is the most biologically active in humans. Vitamin E is found in nuts, seeds, vegetable oils, and green leafy vegetables such as spinach and broccoli [162]. Well known for its anti-oxidative role, vitamin E has been associated with versatile health promotion and additive effects in treatments for many diseases, including cardiovascular, hepatic, and Alzheimer’s diseases [163,164,165].

For epileptic patients, 400 IU of daily vitamin E supplementation for three months has been shown to reduce seizure frequency by approximately 60% compared with a placebo group that showed no difference in the number of seizures (Table 3) [14]. Two other clinical studies have confirmed the beneficial effects of chronic vitamin E administration in patients with refractory epilepsy [15,16], supporting the use of vitamin E as an adjunctive therapeutic option for epilepsy. However, the authors of another randomized, double-blind clinical trial have reported that vitamin E supplementation over three months did not affect the occurrence of seizures regardless of the different types of epilepsy (Table 3) [166]. 

Many studies have found an anticonvulsant effect resulting from vitamin E treatment [167,168,169,170,171]. Administered after status epilepticus, it promotes the restoration of glutamate metabolisms by normalizing seizure-induced inhibition of glutamine synthetase (GS), an enzyme that can reduce synaptic glutamate levels [172]. Vitamin E can also inhibit activation of protein kinase C δ [173,174], which can block GS expression [175], leading to the disinhibition of GS and ultimately the reduction of excitotoxicity. Vitamin E treatment can also offer neuroprotective effects against excessive generation of ROS [176], suggesting anti-oxidative effects. Supporting this hypothesis, many studies investigating pre- or co-treatment of vitamin E in animal models of epilepsy have found that vitamin E supplementation can reduce ROS accumulation and attenuate protein and lipid peroxidation [171,173,177,178,179,180]. In relation to inflammation enhanced by acute seizures, vitamin E can downregulate the expression of IL-1β and TNF-α [168,172,177], implying an anti-inflammatory function. 

### 4.4. Vitamin B6

Vitamin B6 is a water-soluble vitamin that plays a vital role in the development and maintenance of the central nervous system [181]. Dietary vitamin B6 is present in fish (tuna and salmon), meat (beef liver, pork, lamb, and chicken), milk products (cheese and yogurt), grains (yeast bread and biscuits), vegetables (carrots, onion, and tomato), and fruits (berry, apple, watermelon, and banana) [181,182]. It has six vitamer chemicals: pyridoxine, pyridoxamine, pyridoxal, pyridoxine 5’-phosphate (PNP), pyridoxamine 5’-phosphate (PMP), and pyridoxal 5’-phosphate (PLP). In general, meat-derived vitamin B6 consists of phosphorylated pyridoxal and pyridoxamine, while plant-derived vitamin B6 contains phosphorylated pyridoxine. 

Once pyridoxine, pyridoxamine, and pyridoxal are transferred to the liver after absorption, these vitamers are phosphorylated by pyridoxal kinase to generate PNP, PMP, and PLP, respectively [183,184]. Additional conversion of PNP and PMP to PLP can occur by pyridoxine-5’-phosphate oxidases (PNPO) to produce a circulatory, active form of vitamin B6 in the blood. As pyridoxal is the only vitamin B6 form that can cross the BBB [185], PLP needs to be hydrolyzed to pyridoxal by tissue non-specific alkaline phosphatase (TNAP). In the neurons, PLP is regenerated by pyridoxal kinases, meaning it can act as an active co-factor [183]. PLP can play a crucial role in the synthesis of multiple neurotransmitters, including GABA, glycine, dopamine, serotonin, and histamine [183]. For example, PLP can convert glutamate to GABA by facilitating the activation of glutamate decarboxylase, suggesting that vitamin B6 deficiency may enhance network excitability in the brain. As we discussed in Section 2.2, pyridoxine deficiency can cause unprovoked seizures which are classified as a type of multifarious epilepsy [3]. 

Vitamin B6 supplementation has been demonstrated to be beneficial in some, but not all, studies evaluating patients with epilepsy (Table 4). In the 1960s, two clinical studies investigated the effects of vitamin B6 administration on the amelioration of seizures. The researchers found that some patients who received vitamin B6 showed significant clinical improvement, although this was not observed for all patients [17,18]. Later, Jiao and colleagues performed a randomized, placebo-controlled trial in which 30 or 50 mg of pyridoxine infusion could resolve the occurrence of recurrent seizures [19]. The total response rate in the pyridoxine-treated group was 92.5%, a significantly higher rate than the control group. However, other clinical studies involving varying types of epilepsy have failed to illustrate anticonvulsive effects following vitamin B6 treatment [186,187,188]. As all of the clinical studies assessing the therapeutic efficacy of vitamin B6 are small-scale studies interpreting mixed results from various epilepsies, more elaborate studies will be necessary to provide conclusive information about the role of vitamin B6 in epilepsy.

Genetic epilepsies associated with vitamin B6 deficiency include PNPO deficiency, infantile hypophosphatasia, pyridoxine-dependent epilepsy, and type II hyperprolinemia [189]. In an attempt to gain molecular insight, PNPO mutations frequently seen in patients with neonatal epileptic encephalopathy were introduced into Chinese hamster ovary cells, and PNPO activities and subsequent PLP synthesis both declined [190,191], suggesting PNPO-mediated disruption of vitamin B6 synthesis. Infantile hypophosphatasia features a mutation in TNAP, a critical enzyme for the cellular transport of PLP [192]. Indeed, TNAP-deleted mice have displayed increased serum PLP levels due to the reduced availability of cellular PLP and the development of spontaneous seizures with a decrease in total GABA [193]. Moreover, pyridoxine and pyridoxal administration have been found to rescue the seizure phenotype of TNAP mutant mice, providing a molecular mechanism for hypophosphatasia-induced seizures. Pyridoxine-dependent epilepsy is an autosomal recessive disorder with an ALDH7A1 mutation [20,194]. ALDH7A1 encodes α-aminoadipic semialdehyde dehydrogenase, also known as antiquitin, which generates α-aminoadipic acid in the process of lysine catabolism. ALDH7A1 mutation induces antiquitin deficiency, which results in elevated levels of α-aminoadipic semialdehyde [194,195]. This can further promote the accumulation of piperidine-6 carboxylate (P6C), which can inactivate PLP by forming a Knoevenagel condensation product [196]. In a zebrafish model of ALDH7A1 deficiency, these reactions were found to lead to an overall reduction of available PLP in the brain and subsequently disrupt conversion of glutamate to GABA, generating spontaneous recurrent seizures [197]. Type II hyperprolinemia is characterized by the mutation of pyrroline-5’-carboxylate dehydrogenase (P5CDH) [20]. P5CDH deficiency can block the conversion of pyrroline-5’-carboxylate (P5C) to glutamate, causing the accumulation of P5C in the blood. As P5C can trap PLP, similar to PLP consumption by P6C, a P5CDH mutation can deplete the available PLP in the brain, causing epilepsy.

### 4.5. Nutrients Potentially Beneficial for Refractory Epilepsy

Vitamin C (also known as L-ascorbic acid) is a water-soluble vitamin that cannot be endogenously synthesized in the human body [198]. Thus, vitamin C needs to be obtained from foods such as citrus fruits (orange and grapefruit), kiwifruits, strawberries, cantaloupes, and certain vegetables (broccoli, tomatoes, brussels sprouts, and red and green peppers) [198]. Vitamin C can act as an antioxidant, scavenging free radicals to protect cells from ROS-associated damages [199]. Moreover, vitamin C can serve as a co-factor in the enzymatic conversion of dopamine to norepinephrine, regulating neurotransmission [200]. Several animal studies have demonstrated the anticonvulsive effects of vitamin C, which were seen via the reduction of oxidative stress regardless of model systems, including pilocarpine-, pentylenetetrazol-, penicillin-, or kainic acid-induced epilepsy [180,201,202,203,204]. However, even with supporting evidence that patients with epilepsy have shown reduced levels of serum vitamin C [205], no clinical study reporting on the efficacy of vitamin C interventions in epilepsy exists. Currently, only a phase II clinical trial to test the effects of adding vitamin C within the treatment of idiopathic epilepsy (NCT02369822) is ongoing, indicating the requirement of more efforts to elucidate the roles of vitamin C in epilepsy.

Pyruvate is a crucial intermediate in glucose metabolism. Under aerobic conditions, pyruvate can be converted into acetyl coenzyme A, which enters the Krebs cycle to generate adenosine triphosphates, a cellular energy source. Pyruvate can also remove hydrogen peroxides, working as a potent antioxidant [206]. Moreover, a simple derivative of pyruvic acid, ethyl pyruvate can efficiently suppress inflammation [207], suggesting possible therapeutic benefits for epilepsy. Indeed, oral pyruvate administration could significantly reduce the number of spontaneous recurrent seizures in multiple animal models of epilepsy [208]. In addition, a combination therapy of pyruvate, vitamin C, and vitamin E could reduce the formation of seizures [180]. Taken together, these data support the hypothesis that pyruvate may be a novel metabolic target able to be used to treat acquired epilepsies. Based on these preclinical results, clinical trials assessing the therapeutic efficacy of pyruvate will be necessary for the development of new nutrients beneficial for epilepsy.

## 5. Conclusions

Since phenobarbital became known for its anticonvulsive properties in 1912, numerous antiepileptic drugs have been developed [209]. In spite of a wider array of available options in drug selection today, the incidence of refractory epilepsy remains still high, corresponding to 30% of all epilepsy patients. Dietary and nutritional supplements can help address this gap until innovative therapeutic methods are established. In this review, we have provided an overview of epilepsy and its fundamental molecular mechanisms, followed by an introduction to several functional nutrients which have the potential to manage seizure frequencies, i.e., omega-3 PUFA, vitamin D3, vitamin E, vitamin B6, vitamin C, and pyruvate. Despite slightly favorable efficacy having been reported in general, there is still controversy surrounding the therapeutic benefits of functional nutrients within epilepsy. We speculate that these discrepancies may come from the inclusion of various epilepsies with different etiologic backgrounds, varied durations of, and different dose regimens and treatment durations of functional nutrients. Thus, more elaborate clinical trials will be required for the accurate evaluation of nutritional supplements for epilepsy, which will enable an informative meta-analysis showing higher levels of evidence to be undertaken in future. Currently, limited dietary and nutritional consideration by patients with epilepsy can be undertaken due to insufficient research attention, which is surprising given the extensive investigative efforts devoted to other diseases, such as Alzheimer’s and Parkinson’s disease and stroke [210,211,212,213]. As experimental evidence regarding putative candidate nutrients in animal models of epilepsy [214,215,216,217] is readily available, it will be necessary to examine the translational potential of these molecules to facilitate the development of nutraceuticals for epilepsy treatment. Finally, because of a study in which megavitamin therapy of pyridoxine was shown to cause neurotoxic effects [218], a cautionary note must be included regarding the optimal dose of functional nutrients. 

## Figures and Tables

**Table 1:** Clinical studies assessing the efficacy of fish oil in patients with epilepsy.

Author	Number of Patients	Age (years)	Study Type	Intervention		Duration	Any Other Intervention	Final Result
				Experimental (Exp)	Control (Con)			
Schlanger et al., 2002 [4]	*n* = 5	Range: 12–26	Observational	5 g DHA, EPA, ALA	-	24 weeks	Anticonvulsive drugs	Decreased seizures after intervention
Yuen et al., 2005 [5]	*n* = 57 (Exp: 13, Con: 22)	Range: 19–65	Randomized, double-blind, placebo-controlled trial	1000 mg fish oil (171 mg EPA, 112 mg DHA, <100 IU Vit A, <40 IU Vit D)	Placebo (70% palm olein, 15% rapeseed oil, 15% sunflower oil)	12 weeks	Antiepileptic drugs	First 6 weeks: five had 50% reduction in seizures Second 6 weeks: no difference
Puri et al., 2007 [106]	*n* = 7 (Exp: 3, Con: 4)	Mean ± SD: 50.7 ± 13.6 (Exp), 40.5 ± 12.0 (Con)	Randomized, double-blind, placebo-controlled trial	1000 mg fish oil (171 mg EPA, 112 mg DHA, <100 IU Vit A, <40 IU Vit D)	Placebo (70% palm olein, 15% rapeseed oil, 15% sunflower oil)	12 weeks	-	No significant correlations between seizures and changes in spectroscopic resonances
Dahlin et al., 2007 [107]	*n* = 25	Range: 1.5–18.1	Prospective cohort	1–2 g fish oil with meal, four times/day	-	48 weeks	Antiepileptic drugs	No effect on seizures
Bromfield et al., 2008 [108]	*n* = 21, (Exp: 12, Con: 9)	Range: 25–55 (Exp), 22–62 (Con)	Randomized, double-blind, placebo-controlled trial	2.2 mg EPA and DHA (3:2 ratio) 1.1 g/day (1 week) → 1.1 g, two times/day (3 weeks)	Mineral oil	4 weeks	Antiepileptic drugs	No effect on seizures
DeGiorgio et al., 2008 [10]	*n* = 11	Range: 18–65	Randomized, double-blind, two periods crossover clinical trial, 6 weeks washout period	1200 mg fish oil/day (216 mg EPA, 144 mg DHA)	Soybean oil 8 capsules/day	30 weeks	Antiepileptic drugs	No effect on seizures Restoration of heart rate variability
Al Khayat et al., 2010 [6]	*n* = 20	Range: 3–10	Observational	1000 mg PUFA (700 mg DHA and 300 mg EPA)		6 months	Antiepileptic drugs	Decreased seizure frequency, seizure duration and seizure severity after intervention
Yuen et al., 2012 [109]	*n* = 10	Range: 23–75	Observational	500 mg EPA with 10 mg mixed tocopherols/capsule, 2 capsules/day	-	12 weeks	Antiepileptic drugs	No effect on seizures Reduced seizure severity in one person
DeGiorgio et al., 2015 [7]	*n* = 24	Range: 18–56	Randomized, double-blind, three periods crossover clinical trial, twice 6 weeks washout	Fish oil capsule (216 mg EPA, 144 mg DHA (360 mg fatty acids/capsules)) Low-dose group: 1080 mg/day High-dose group: 2160 mg/day	3 corn oil capsules/twice day	42 weeks	Antiepileptic drugs	Decreased seizures in low-dose fish oil
Reda et al., 2015 [8]	*n* = 70	Range: 4–12 Mean ± SD: 6.9 ± 2.5 (Exp), 6.6 ± 2.4 (Con)	Randomized, single-blind trial	1200 mg fish oil (240 mg DHA, 360 mg EPA, Vit E)	Corn oil	12 weeks	Antiepileptic drugs	Elevated the seizure threshold in fish oil supplementation group
Omrani et al., 2019 [9]	*n* = 50	Range: 18–55	Randomized, triple-blind, placebo-controlled trial	Omega-3 fatty acids capsules (120 mg DHA, 180 mg EPA plus Vit E)	Placebo capsule	16 weeks (two times/ day)	Antiepileptic drugs in refractory epilepsy	Decreased seizure frequency and duration in omega-3 fatty acids supplementation group

Legend: DHA, docosahexaenoic acid; EPA, eicosapentaenoic acid; ALA, alpha-linolenic acid; PUFAs, polyunsaturated fatty acids; Vit, vitamin; -, not indicated; IU, international unit.

**Table 2:** Clinical studies assessing the efficacy of vitamin D in patients with epilepsy.

Author	Number of Patients	Age (Years)	Study Type	Subgroup		Duration	Any Other Intervention	Final Results
				PWE	Control (Con)			
Christiansen et al., 1974 [11]	*n* = 23	Range: 6–27	Observational	A: N = 9, 4000 IU, 16,000 IU/day Vit D3 B: N = 14, 8000 IU/day Vit D3		84 days	Antiepileptic drugs	Reduction in the number of seizures after intervention
Hollo et al., 2012 [12]	*n* = 13	Range: 19–60	Observational	Vit D3		90 days	-	40% decrease in seizures after intervention
Tombini et al., 2018 [13]	*n* = 202 (PWE: 160, Con: 42)	Mean ± SD: 50.6 ± 19.3	Case-sectional cohort	Cholecalciferol 100,000 IU/week (Vit D deficiency) Cholecalciferol 100,000 IU/2 weeks (Vit D insufficiency)	Cholecalciferol 100,000 IU/week (Vit D deficiency) Cholecalciferol 100,000 IU/2 weeks (Vit D insufficiency)	3 months	Antiepileptic drugs	PWE showed low Vit D No effect on drug-resistant seizures

Legend: PWE, patients with epilepsy; -, not indicated.

**Table 3:** Clinical studies assessing the efficacy of vitamin E in patients with epilepsy.

Author	Number of Patients	Age (years)	Study Type	Intervention		Duration	Any Other Intervention	Final Results
				Experimental (Exp)	Control (Con)			
Ogunmekan et al., 1989 [14]	*n* = 24 (Exp: 12, Con: 12)	Range: 6–17	Randomized, double-blind, placebo-controlled clinical trial	Vit E	Placebo	3 months	Antiepileptic drugs	>60% reduction in seizure frequency
Hom et al., 1991 [15]	*n* = 52 (Exp: 27, Con: 25)	-	Randomized, double-blind, placebo-controlled trial	Vit E	Placebo	3 months	Antiepileptic drugs	Reduction in seizure frequency: 30% of all patients Reduction in seizure frequency: 58% of medically stable patients
Raju et al., 1994 [166]	*n* = 43	-	Randomized, double-blind, two periods crossover trial	Vit E and then placebo	Placebo and then Vit E	6 months (for two treatments)	Antiepileptic drugs	No significant change in seizure frequency
Mehvari et al., 2016 [16]	*n* = 65 (Exp: 32, Con: 33)	Mean ± SD: 28.8 ± 5.3 (Exp), 28.6 ± 8.8 (Con)	Randomized, double-blind, placebo-controlled trial	400 IU/day Vit E	Placebo	6 months	Antiepileptic drugs	Reduction in seizure frequency Improved EEG findings

Legend: EEG, electroencephalogram; -, not indicated.

**Table 4:** Clinical studies assessing the efficacy of vitamin B6 in patients with epilepsy.

Author	Number of Patients	Age (years)	Study Type	Intervention		Duration	Any Other Intervention	Final Result
				Experimental (Exp)	Control (Con)			
Fox and Tullidge, 1946 [186]	*n* = 8	Range: 14–15	Observational	Four patients: 100 mg pyridoxine/day for 3 weeks and then 100 mg pyridoxine/day for 4 weeks Two patients: 20 mg/day for 4 weeks and then 100 mg pyridoxine/day for 4 weeks Two patients: only 20 mg/day for 8 weeks	-	7–8 weeks	-	No effects
Livingston et al., 1955 [187]	*n* = 31	Range: 0.5–14	Observational	20 mg (two times/day); pyridoxine dosage was increased to 100 mg/day	-	At least 1 mo	Antiepileptic drugs	Pyridoxine failed to control seizures Larger doses of pyridoxine increased the number of seizures
Hagberg et al., 1964 [17]	*n* = 3	Range: 1–3	Observational	One patient: 60 mg Vit B6 Two patients: 160 mg Vit B6	-	NR	Antiepileptic drugs	Improved EEG findings, normal development, decreased mental changes, reduced dosage of anticonvulsive drugs
Hansson and Hagberg, 1968 [18]	*n* = 56	Children	Observational	160–300 mg pyridoxine		At least 6 weeks	Antiepileptic drugs	Five patients showed significant clinical improvement; side effects for only one patient
Heeley et al., 1968 [188]	*n* = 70	Range: 0.2–11	Observational	30 mg/day pyridoxine	-	1–4 weeks	Antiepileptic drugs	No clinical improvement
Jiao et al., 1997 [19]	*n* = 90, (Exp: 40, Con: 50)	Range: 0.1–12	Randomized, controlled trial	30 or 50 mg/day pyridoxine	No treatments	NR	Antiepileptic drugs	Recurrent seizures were resolved

Legend: NR, not reported; -, not indicated; mo, month.
